# The Nursing Supplement

**Published:** 1890-02-22

**Authors:** 


### "The Hospital" Nursing Mirror.

Being the Extra Nursing Supplement of "The Hospital" Newspaper.

All Contributions for this Supplement should be addressed to the Editor, The Hospital, 140, Strand, London, W.C., and should have the worl "Nursing" plainly written in left-hand top corner of the envelope.

### En Passant.

INFIRMARY NURSES AND THE PENSION FUND.—Last week a small meeting was held at Paddington Infirmary, when Mr. Burdett explained to the nurses assembled the best way of making use of the Pension Fund. Table F, which secures both sick pay and a pension, was specially recommended to nurses. Three points brought forward in the discussion which followed the address were: (1) In case of marriage, the money paid into the pension fund can be withdrawn to buy a trousseau, or the nurse can continue to subscribe after marriage; (2) in case of death the money is paid to the heirs, or to any person named by the nurse as her chosen successor on the printed paper which accompanies each policy; (3) The returnable premium plan of this Fund enables nurses to put their money in, and subject to reasonable restrictions to withdraw it how and when they like. Arrangements are in progress to allow three per cent. on money so saved, which will be ½ per cent. higher than the Post Office rate.

SHORT ITEMS.—Mr. H. Huxley, the only unmarried son of Professor Huxley, has just become engaged to a lady who, though belonging to a wealthy and well-known Yorkshire family, has been occupying herself lately as a nurse at St. Bartholomew's Hospital.—Miss Agnes Jackson, of Edinburgh Royal Infirmary, has won prizes for instrument work, bandaging, and the best written examination paper.—Lady Audrey Buller is organizing district nursing at Crediton.—There are three vacancies in the Bristol House of Help; the charge is £40 a year. It is a kind of sisterhood.—A registry office for nurses has been opened in Sweden by the Frederika Bremer Trustees.—The nurses of the Hospital for Sick Children, Great Ormond Street, sent Mr. Toole a horseshoe of violets before his departure for Australia, in recognition of many kindly acts.—In future the probationers of Newcastle Infirmary will receive no salary the first year, and only £10 the second.—Miss Bassett, late Sister Mary at Guy's, is doing well with her nursing institute at Biarritz.

DISTRICT NURSING AT GLASBURY.—On the afternoon and evening of Friday, January 24th, highly-successful amateur theatrical performances took place in the village of Glasbury, Radnorshire, in aid of the fund for maintaining a trained midwife-nurse for the sick poor in Glasbury and the adjoining parishes of Llowes and Boughrood. The plays selected for representation were *The Coming Woman* and *Marjory Daw*. A nurse has been at work in this district for nearly a year, and many serious cases have received the benefit of her services. During the past three months alone 326 visits have been paid by the nurse, which in a scattered country parish, or rather parishes, represents a good deal of work. Additional funds are required in order to carry on this good work in a spot where such help is much needed, the nearest medical man being at a distance of nearly four miles, and it is to be hoped that some of the generous friends to the cause of nursing the sick poor will come forward and aid the committee in their endeavours. The basis of the work is unsectarian, Church people and Nonconformists alike being entitled to the services of the nurse. The district is a poor one, the inhabitants being principally agricultural labourers, whose wages are too small to admit of their paying much, if anything, for the nurse; therefore, the Nursing Fund is entirely dependent on the voluntary subscriptions and donations of the well-to-do. Any aid will be gratefully acknowledged by Mrs. Battiscombe, Woodlands, Glasbury, Radnorshire, R.S.O., hon. sec. to the Glasbury Nursing Fund.

TIRED AMERICAN NURSES.—We learn from a New York paper that the Training School for Nurses attached to Bellevue Hospital has just been made the recipient of two acres of land with buildings thereon at Bell Island, Norwalk Harbour. This gift came from Hugh O. Northcote, a son of Sir Stafford Northcote, in recognition of the faithful services rendered by these nurses to his wife during her last illness. This plot of land is intended for a summer retreat where the tired out nurse may regain health and strength after her arduous duties at the bedside. It is a free gift, the transfer being made by a formally executed deed to the Training School.

NURSES FOR THE POOR.—An effort is being made to establish a home for nurses in the poor districts of Haggerstone and Hoxton. These nurses are ladies who have received full hospital training, and an additional special training in district nursing at the Central Home of the Metropolitan National Nursing Association, Bloomsbury Square, W.C. The sick poor receive in their own homes the services of the nurses, free of charge, the association being on the same footing as the hospitals, supported entirely by voluntary contributions. Funds are greatly needed to help in this good work, also gifts of old linen, clothing, medical and surgical appliances and surgical aid letters, will be gratefully received by Mrs. R. Franks, hon. secretary, 35, Nichols Square, Hackney Road, N.E.

THOUSANDS OF POUNDS FOR THE NATIONAL PENSION FUND.—We have the pleasure to acknowledge the following sums towards the £14,000 now being raised to at once bring the Donation Bonus Fund up to £40,000. We hope to be able to acknowledge contributions amounting to £1,000 every week until the £40,000 are completed. Contributions of any amount may be sent to the Editor of The Hospital, or to the Manager, National Pension Fund for Nurses, 8, King Street, Cheapside, London, E.C.:

The Fifth Thousand.

| | £ | s. | d. |
|---|---|---|---|
| Messrs. Linton, Clarke, and Co. ... | £100 | 0 | 0 |
| Messrs. Quilter, Balfour, and Co. ... | 100 | 0 | 0 |
| Messrs. Ricardo and Co. ... ... ... | 100 | 0 | 0 |
| Messrs. Steer, Lawford, and Co. ... | 100 | 0 | 0 |
| Messrs. Williams and Meyer ... ... | 100 | 0 | 0 |
| Mr. Wm. Trotter ... ... ... ... ... | 100 | 0 | 0 |
| Messrs. A. Mocatta and Co. ... ... | 100 | 0 | 0 |
| Messrs. Wedd, Jefferson, and Co. ... | 52 | 10 | 0 |
| Messrs. Garett, Sons, and Co. ... ... | 26 | 5 | 0 |
| Mr. H. K. Ricardo ... ... ... | 25 | 0 | 0 |
| Mr. Murray Griffith ... ... ... | 25 | 0 | 0 |
| Mr. F. W. Lawrence ... ... ... | 25 | 6 | 0 |
| Messrs. Smithers and Williams ... ... | 21 | 0 | 0 |
| Mr. Levi Cohen ... ... ... ... | 10 | 10 | 0 |
| Messrs. Crocker Bros. ... ... ... | 10 | 10 | 0 |
| Mr. F. H. Durlacher ... ... ... | 10 | 10 | 0 |
| Messrs. Hilder and Moens ... ... | 10 | 10 | 0 |
| Messrs. Price Bros. ... ... ... | 10 | 10 | 0 |
| Messrs. A. Thomson and Co. ... ... | 10 | 10 | 0 |
| Mr. C. J. Whittington ... ... ... | 10 | 10 | 0 |
| Messrs. Schillizzi and Co. ... ... | 10 | 10 | 0 |
| Messrs. Hensley and Aston ... ... | 10 | 10 | 0 |
| Mr. G. Fletcher ... ... ... ... | 5 | 5 | 0 |
| Messrs. Francis and Praed ... ... | 5 | 5 | 0 |
| Mr. C. A. Cresswell ... ... ... | 5 | 0 | 0 |
| Mr. T. Kitchin ... ... ... ... | 5 | 0 | 0 |
| Mr. C. R. Fry ... ... ... ... | 5 | 0 | 0 |
| Mr. T. Quihampton ... ... ... | 2 | 2 | 0 |
| Mr. H, G. Fitclarance ... ... ... | 1 | 1 | 0 |
| Mr. A. G. Kitching ... ... ... | 1 | 1 | 0 |
| Mr. H. Slade ... ... ... ... | 1 | 1 | 0 |

Amount already received, **Five Thousand Pounds.**

### Nursing Lectures.

By Percy Lewis, M.D.

### Lecture III.—SYMPTOMS IN CHEST DISEASES.

Cough is generally present in chest diseases, it is, in fact, for cough that a patient often first comes to the hospital. The physician's duty is to find out the cause, and lay down the appropriate treatment; the nurse's duty is to note the presence or absence, frequency and character; whether it keeps the patient awake; whether the patient's account of it is accurate or not, and whether it comes on at any special time; if asked, to give her opinion as to whether it is better or worse from day to day. Patients often state that they have no cough because it is so slight that they become accustomed to it. Sometimes cough occurs only on waking in the morning; at other times only on lying down or going to bed. The character of a cough varies; it may be dry, moist, loud, clanging, brassy, or barking. It may occur only in paroxysms, only after meals, or in particular positions. It may be associated with retching or vomiting, with crowing breathing or bleeding from the nose, eyes, and ears, as in whooping-cough. The object of cough is to get rid of irritation or abnormal accumulation somewhere in the throat, air tubes, or chest. The commonest cause is excessive secretion of mucus, "sputum," or "expectoration," as it is called. In every case the sputum must be kept for the physician to see at his next visit. After he has once seen it a little strong carbolic acid solution or some other antiseptic must be kept constantly in the spittoon.

Expectoration has to be noted as to whether it contains blood or not; whether frothy when first coughed up, becoming watery only on standing; whether it has any smell; how much is expectorated during the day; and whether in small or large quantities. Nurses should endeavour to be extremely accurate in all measurements. I have often noticed that amounts of sputum are always reported as being six, seven, or eight ounces, never six-and-a-half or seven-and-three-quarters. Should you notice any abnormal colour suggestive of tobacco, sweets, or other forbidden articles, you will at once institute a search of the patient's bed and locker, in order to prevent any further harm resulting from his indulging in them, and should you be successful, report it. In pneumonia, the sputum is described as "rusty," or "prune juice." This should be shown to the physician if it occurs.

The normal rate of breathing varies from twelve to twenty respirations per minute. When there is any difficulty of breathing the rate becomes materially altered. Dyspnœa, or difficulty of breathing, is a common and most important symptom in the class of diseases we are now considering. It may depend on a variety of causes, as in cases of heart, lung, or kidney affections. Nurses must note its presence, and be able to describe it as it occurs during the absence of the physician. You have heard of nurses being able to tell by a patient's breathing whether he was awake or asleep. That is a feat which may now seem to you very difficult, although with practice and continual observation you may very soon attain to it.

Dyspnœa may be continuous or come on in definite attacks. Learn to count the respirations per minute. In bad cases do this often and keep a note of it. Do not count them just after an attack of coughing, as this will give an erroneous idea of their rapidity, and try not to let the patient know you are counting, or he at once alters the rate. We get over this difficulty by holding the hand and pretending to count the pulse, while really watching the breathing. Note whether the breathing is accompanied by any abnormal sounds, such as gurgling, crowing, or wheezing. If the dyspnœa occurs in paroxysms, observe how often they come on, if they are brought on by anything special, the position assumed by the patient during the attacks, whether they are accompanied by much distress or sense of suffocation, and whether they come on suddenly or gradually. Often when the dyspnœa is bad, especially in heart and aneurism cases, it is impossible for the patient to lie down; he may, indeed, be compelled to sit up for days and nights together, even a semi-recumbent posture inducing a sense of impending dissolution. In worse cases still, they cannot even remain in bed, but spend all their time in a chair, in order to give their chests the freest play possible.

The rhythm of breathing may be altered, being increased or slowed and interspersed with sighs, or alternately quick and slow, changing every few minutes, as is observed in some hysterical conditions.

It is important that you should be able to recognise what is called "Cheyne Stokes" breathing, which consists in the occurrence of a series of inspirations increasing to a maximum and then declining in force and length until a state is produced when apparently no breathing is taking place.

In asthma patients are often ordered an inhalation or a capsule, or a draught, to be taken at the commencement of an attack. Now, it is extremely important that the remedy should be ready to be applied at a moment's notice. Often the attacks can be cut short if the remedy is given directly the patient begins to feel difficulty of breathing, but when once the attack is well established the remedy may be found useless.

Cyanosis is a condition depending on some interference with respiration or circulation. By cyanosis is meant lividity, duskiness, or blueness of the face, or, indeed, of any part, but the face shows it most. Its duration and degree must be noticed, for it may only come on with an asthmatical or coughing attack, or, on the other hand, may exist for days. People when under the influence of laughing gas always exhibit more or less of it.

Pain in any disease is always to be reported, and without giving your opinion as to the cause. Pain may be of different kinds and degrees; it may be described as sharp, shooting, aching, stabbing, catching, dull, griping, throbbing, etc.

When a patient spits up blood, he is said to have hæmoptysis. The amount may vary from a few isolated streaks, to a rush of blood so large that the patient dies in a moment. When in any quantity, it is all important to note whether it is vomited or coughed up. This helps us to determine whether it comes from the chest or stomach. The relations of a nurse to a case of hæmoptysis are most important. First of all, and this applies equally to all cases, never appear flurried or anxious, but be firm and matter-of-fact. In default of orders to the contrary, the patient must be kept perfectly still and quiet. He must not be allowed to raise himself or make the simplest exertion without the permission of the doctor. The nurse on duty must never let him out of her sight for a minute, and should he be one of those excitable people, she must sit by him and do everything in her power to keep him quiet. These people take advantage of any relaxation on the part of the nurse, for they are often slow to appreciate the extreme danger of their position. Any remedies ordered to be given in case of hæmoptysis coming on, must always be kept close at hand, not a moment is to be lost when the attack occurs. If, for instance, it is a hypodermic injection which is to be given, one nurse will go for the house surgeon, while another will be getting the syringe out of its case and filling it with the solution; a second or two lost may make all the difference between life and death.

Palpitation or irregular action of the heart occurs in a variety of diseases, chiefly, however, as might be expected, in heart diseases. It can easily be felt by placing your hand over the patient's heart. Remedies to be given when the attack comes on should be kept where they can be quickly obtained.

### The Nurses' Bookshelf.

### DISTRICT NURSING.*

Mrs. Dacre Craven has filled a great want in the nursing world by writing this book. Whereas books on home and hospital nursing have been multiplying beyond all need lately, no book existed on district nursing till Mrs. Craven took up her pen. The district nurse has a certain training of her own; she has to be instructed in the art of doing without, and the truly skilful workman is ever the one who does the best work with the poorest tools. In the book before us are directions for extemporising outside blinds, bed-rests, bronchitis kettles, filters, and a hundred more small comforts which the sick poor too often have to go without. The nurse is taught how to use her own common sense; she is not crammed with technical details, but is shown how to turn everything to good use; how to behave so as to be welcome to the poor; and how to keep her books to the satisfaction of the committee. A district nurse is gradually becoming a necessary institution in every village and town, and frequent are the queries we receive about the duties and benefits of such a woman. In future we can refer all correspondents to this book, where they will find the subject fully and skilfully dealt with, and will find specimen tables of lists of cases, rules for the nurse, and other interesting information. To district nurses the book will be simply invaluable; and private nurses can learn much from its pages. Many small households to which the private nurse finds her way, would be truly grateful if the nurse made fewer demands for appliances, and knew how to make a bed cradle with corkscrews; how to make ice-drinks without ice, and other strange devices taught in this clever book.

### NURSING FOR SIXPENCE.†

The two best points about this book are the price and the portrait of the authoress in the commencement. It is a wonderful sixpennyworth, containing a great deal of information of the usual kind given in these numerous cheap handbooks on nursing. We should have been better pleased if Mena Drew had not gone out of her way to advertise electropathic belts, patent foods, and other articles.

### NURSING QUESTIONS AND ANSWERS.‡

Here again is a useful book which supplies a long-felt want. The poor trained nurse of modern days has to study hard, as well as work hard, and must pass an examination if she desires to rise in her profession. It is one thing to be a good and faithful nurse, and quite another thing to be able to talk or write well on the subject. Yet surely a certain facility might be gained by reading this book on nursing written in the question and answer method? It would acquaint a nervous nurse with the sort of ordeal before her, and accustom her to the style of queries likely to be asked. For matrons and sisters who desire to find out how much probationers know, and for those whose duty it is to examine probationers, this book is sure to prove useful. The book has been written specially for the pupils of the nursing classes given by the St. John's Ambulance Association, but is so complete and correct it can be recommended to those who mean to become trained nurses.

* A Guide to District Nurses. By Mrs. Dacre Craven. (Macmillan and Co.)

† Hints on Nursing, by Mena Drew. (R. Forder, London.) Price 6d.

‡ Questions and Answers on Nursing. By J. W. Martin, M.D. (Bailliere, Tindall, and Cox.) 1s. 6d.

### Everybody's Opinion.

*[Correspondence on all subjects is invited, but we cannot in any way be responsible for the opinions expressed by our correspondents. No communications can be entertained if the name and address of the correspondent is not given, or unless one side of the paper only be written on.]*

### MEUM AND TUUM.

Justine writes: I see you comment upon the non-distribution of "Truth's" toys at the Lewisham Workhouse, and you say the difference between *meum* and *tuum* is hard to impress on certain minds, the general belief being that in public institutions the inmates have no right to possess aught of their own. Now, I go further than this, and complain that inmates of public institutions, so far as from being allowed to have anything of their own are not even permitted to be anybody. They become "No. 10" or "No. 12," as the case may be—that's all. This is particularly hard when it happens to a nurse—ill and warded in a strange hospital. The other day I found myself compelled to undergo an operation, and I was so weary and worn out with sick nursing (I have been a hospital trained nurse these fifteen years) that when my kind surgeon offers to take me into the hospital at a moment's notice I gladly accept. Imagine my disgust when I suddenly realize that I am no longer to have an idea of my own, and that if I do venture to pass a remark the probationer answers me with such an air of authority that I, in my weakness, feel inclined to burst into tears. Now why should a nurse, if she has the misfortune to be ill, be treated as a nonentity? Surely a kind word from her sisters in the profession would only help her to bear her trial. But, more than this—how can nurses calmly and unblushingly tell their patients untruths in order, as they falsely think, "to quiet them," when the truth frankly told would be far more bravely borne by the sufferer? I could not see my wound, but knowing well a stitch was still left behind causing irritation, I politely asked the dresser if all the stitches were removed? "Yes," I was told. During that night I sat up in bed, prepared myself with a sharp pair of scissors intending to remove the offending stitch, when the kindness of my good surgeon flashed across my mind, my naughty thought fled, and the dresser is left the pleasure of confirming yesterday's untruth. A friend of mine, also a nurse, was ill a short time since; the drainage-tube fell out of place, she informed her nurse of the fact, but the nurse emphatically declared it had done no such thing, although it remained out for hours.

### A FIRST OPERATION CASE.

H. R. E. writes: I shall remember my first operation case as long as I have any recollection of my hospital life and work. I was trained at the Liverpool Royal Infirmary. My period as a probationer commenced in 1875, for three years' training. Before I had finished my first year I was put in charge of a medical ward. At that time the infirmary was undergoing the process of cleaning-down, white-washing, &c. To allow the surgical wards to be first cleaned, the cases were sent up to the medical wards. It was my duty at that time to relieve a patient with an anuerism under the knee. The patient was a fine, strong, healthy man—and to look at him no one would have thought he was a patient, and to undergo an operation. The man had been in the infirmary a few days, when a consultation of physicans and surgeons was held, and it was decided to remove the anuerism and to save the limb. The patient was told the decision of the doctors, and he seemed very thankful to know his leg would be spared and not cut off. The next day the man was taken to the theatre, and, under the hands of the doctors and the eyes of 150 students, the anuerism was removed. He was brought back to the ward, and all appeared to be going on satisfactorily. Watched well by doctors, students, and nurses, myself included, the poor fellow said he would soon be all right, and able to resume his work again. But, alas! gangrene set in very suddenly, and in less than twenty-four hours from the first operation a second one took place, and the leg cut off above the knee, the doctors hoping to save the man's life. The poor fellow pleaded to the doctors to save him for his motherless little ones—a boy and girl. "Save me, doctors," he cried, "for my poor children's sake." Poor man; his hours were numbered, for in less than four hours after the second operation he was dead. The doctors comforted him by telling him should anything happen to him his children would be put into some kind orphanage home. It is now some time since I left hospital life, but not hospital work. I have been a district nurse, and am at present a private nurse, and I have seen much suffering among the rich as well as among the poor, but I do not remember a case worse than the poor patient I have written about.

### Death in our Ranks.

The death of Nurse Porter on February 10th, at Edinburgh Royal Infirmary, removes one of the most revered of Scotch nurses from the land. Nurse Porter had worked under Syme, Lister, and all the great Edinburgh surgeons of the last 50 years. Her devotion to duty was unflagging, and she died in her uniform while sitting down to rest after helping to dress a patient. All the medical papers have published obituary notices of Nurse Porter.

The death is also announced of Sister Ellen Mary, Mother Superior of St. Mary's Church of England Priory, New Kent Road, but more directly associated with the Workgirls' Protection Society. During her career as a sister for twenty-three years she passed through nearly every phase of charitable work—as a lady nurse in hospital, a teacher of young orphan children, and trainer of young girls; but it was in visiting the sick poor that Miss Ansell excelled.

### Notes and Queries.

### Queries.

(52) *Respirators.*—Where can the Ori-nasal respirators be obtained, and the price? Also the manifold respirator?—*Mona.*

(53) *Going to the Colonies.*—Is there any central office to which I can apply for a post as nurse to an invalid going to the colonies or as matron to women emigrants?—*C.*

(54) *Lunatic Asylums.*—Where can I obtain list of lunatic asylums? Also what hospitals take male nurses, and what qualifications are required to procure situations in same?—*A. B. C.*

### Answers.

*Prescribing.*—We beg all our correspondents to distinctly note that we do not prescribe for our readers, and that queries relating to their various ailments cannot be answered.

(54) *Lunatic Asylums.*—A list will be found in "Burdett's Hospital Annual," price 2s. 6d., from our publishers. The Hamilton Association, of 57, Park Street, London, W., trains male nurses, so do some of the workhouse infirmaries. Qualifications, a good character, and between 20 and 30 years of age.

(53) *Going to the Colonies.*—There is no single office where enquiries could be made. The best course to take would be to write to the Agents-General for the Colonies of Victoria, New South Wales, South Australia, Tasmania, New Zealand, etc.; the High Commissioner for Canada; and the Crown Agents for the Colonies, Downing Street. All the Agents-General nearly have their offices in Victoria Street, Westminster.

*McArthur.*—Hot water, soap, and a rough flannel. See article on Physiology in The Hospital for July 30th, 1889.

(49) *Nursing in Australia.*—In the back numbers of the Nursing Mirror full information is given concerning Australian hospitals and nursing there. A letter from Australia appears once a month in our columns.

(44) *Home for Epileptic Girl.*—Apply to Miss Wicksteed, St. John's Road, Watford.

(47) *Home for Nurse.*—Nurse Leah writes: "I return you many thanks for your kindness in publishing my query and forwarding the answers. I have found what I require."

*Nurse George.*—The address of the association will shortly be published in these pages.

*Miss Bramley.*—See "Reductio ad Absurdum" in the Mirror for January 25th.

*Miss Greenwood.*—The lectures to ladies at the Sanitary Institute commence at three p.m.

*C. L.*—See the article "How to Become a Nurse" in The Hospital for August 31st, 1889.

"*One who wishes fair play to our admirable Hospital Nurses*" is requested to send name and address.

### Appointments.

Bucks Infirmary.—Mrs. Heatley, who has been acting as *locum tenens* since June, has been unanimously elected matron of this infirmary.

St. Thomas's Hospital.—It is with the greatest pleasure we note the appointment of Miss Louisa M. Gordon, as matron, in succession to Miss Pringle, at St. Thomas's. Miss L. M. Gordon is sister of Miss Gordon, of Charing Cross Hospital, and is at present acting as matron of Leeds Infirmary. The contest for the post was very close, and Miss Solly was nearly victorious.

### Vacancies.

The following vacancies are announced:

### Matron.

Islington Infirmary; £80.

Western General Dispensary; £40.

Yeovil Hospital; £30.

### Night-Superintendents.

Cardiff Infirmary; £28.

Queen's Hospital, Birmingham; £30.

### Sister.

Pendlebury Hospital (Fever Ward); £30.

### Nurses.

Aldershot Lock Hospital; £20.
Aimee Nursing Home.
Arbroath Poorhouse; £25.
Bucks Infirmary; £25.
Bangor Nursing Institute; £25.
Beresford House, Beaumont Street, W.
Brixton Institute.
Bournemouth Victoria Home; £25.
Burton-on-Trent Nursing Institution; £25—£28.
Bristol Hospital.
Chester Deaconess House; £24.
Coventry Hospital; £20.
Central Throat and Ear Hospital; £14.
Childrens' Hospital.
Croydon Nurses' Institute.
Fitzroy House, Fitzroy Square; £25.
General Nursing Institute.
Greenwich Union; £20.
Hackney Union; £20.
Hanover Institute; £25.
Isle of Wight; £18.
Jersey Institute; £25.
Liverpool Eye Infirmary; £20.
Mr. Wilson's Institution.
Newcastle Nurses' Home; £22.
Newark Hospital; £18.
Nottingham Association; £25.
Ophthalmic School, Hanwell; £18.
Pendlebury Hospital; £20.
St. Saviour's Infirmary; £20.
St. Vanda's Home, Bromley.
St. Leonard's Nursing Home.
Stoke Nurses' Home (Mental).
Scarborough Institution; £25.
Swansea and South Wales Institute; £25—£30.
Stockton Union; £30.
South Hants Infirmary; £13.
Torquay Institution.
Wimbledon Convalescent Hospital; £22.
West London Hospital; £28.

### District Nurses.

4, Gresham Place, Newcastle; £40.
Great Bedwyn, Wilts; £25.
Hampstead Nursing Association.
Kennington, Ashford; £50.
South London Association.
South London Nursing Association.
St. George's Home, Sheffield.

### Probationers.

Batley Cottage Hospital.
Burton-on-Trent Nursing Institution.
Kendal Hospital; £10.
Notts. Nursing Association.
Nottingham Hospital for Women.
Pembrokeshire Infirmary.
Princess Alice Hospital.
Salisbury General Infirmary.
Southport Infirmary.
Warminster Cottage Hospital.
Weston-Super-Mare Hospital.

### Amusements and Relaxation.

### FOURTH QUARTERLY WORD COMPETITION

**Commenced Jan. 4th, 1890, ends March 29th, 1890.**

Three prizes of 15s., 10s., 5s., will be given for the largest number of words derived from the words set for dissection.

Proper names, abbreviations, foreign words, words of less than four letters, and repetitions are barred; plurals, and past and present participles of verbs, are allowed. Nuttall's *Standard* dictionary only to be used.

N.B.—Word dissections must be sent in WEEKLY, arranged alphabetically, with correct total affixed.

The word for dissection for this, the EIGHTH week of the quarter, being

"ORLEANS."

| Names. | Feb. 13. | Totals. |
|---|---|---|
| Holland | 362 | 618 |
| Red Cape | — | 132 |
| Nurse Marie | 233 | 428 |
| Garfield | | 243 |
| Midget | 343 | 579 |
| E. M. S. | — | 149 |
| Sister | 344 | 591 |
| Duchess | 298 | 413 |
| Ellice | 350 | 623 |
| Reldas | 361 | 644 |
| Jenny Wren | 298 | 565 |
| Mopus | — | 122 |
| Neptune | — | 118 |
| Qu'appelle | 344 | 610 |
| Whitehoe | 322 | 576 |

| Names. | Feb. 13. | Totals. |
|---|---|---|
| Nurse Clague | — | 40 |
| Lauriston | 335 | 620 |
| B. E. T. | 263 | 438 |
| Jacko | — | 110 |
| Lady Clara | | 258 |
| Patience | 364 | 651 |
| Ecila | 366 | 643 |
| Maude | 327 | 605 |
| Esperance | 346 | 628 |
| Ruby | 341 | 614 |
| M. W. | 346 | 628 |
| Crystal Palace | 340 | 568 |
| Jackanapes | 297 | 488 |
| M. M. M. | — | 25 |
| A. F. | 268 | 298 |

### Notice to Correspondents.

N.B.—All letters referring to this page which do not arrive at 140, Strand, London, W.C., by the *first post on Thursdays*, and are not addressed PRIZE EDITOR, will in future be disqualified and disregarded.

